# Can we predict delayed undesirable events after blunt injury to the torso visceral organs?

**DOI:** 10.1002/ams2.330

**Published:** 2018-01-30

**Authors:** Kenichiro Uchida, Yasumitsu Mizobata, Naohiro Hagawa, Tomonori Yamamoto, Shinichiro Kaga, Tomohiro Noda, Naoki Shinyama, Tetsuro Nishimura, Hiromasa Yamamoto

**Affiliations:** ^1^ Department of Traumatology and Critical Care Medicine Osaka City University Graduate School of Medicine Osaka Japan

**Keywords:** Blunt trauma, delayed rupture, non‐operative management, pseudoaneurysm

## Abstract

**Aim:**

Blunt injuries to visceral organs have the potential to lead to delayed pseudoaneurysm formation or organ rupture, but current trauma and surgical guidelines do not recommend repetitive imaging. This study examined the incidence and timing of delayed undesirable events and established advisable timing for follow‐up imaging and appropriate observational admission.

**Methods:**

Patients with blunt splenic (S), liver (L), or kidney (K) injury treated with non‐operative management (NOM) in our institution were included and retrospectively reviewed.

**Results:**

From January 2013 to January 2017, 57 patients were admitted with documented blunt visceral organ injuries and 22 patients were excluded. Of 35 patients (L, 10; S, 17; K, 6; L & S, 1; S & K, 1) treated with NOM, 14 (L, 4; S, 9; K, 1) patients underwent transcatheter arterial embolization. Delayed undesirable events occurred in four patients: three patients with splenic pseudoaneurysm on hospital day 6–7 and one patient with splenic delayed rupture on hospital day 7. The second follow‐up computed tomography scan carried out 1–2 days after admission did not show any significant findings that could help predict undesirable results of delayed events. The patients with delayed events had longer continuous abdominal pain than that of event‐free patients (*P* = 0.04).

**Conclusions:**

Undesirable delayed events were recognized on follow‐up computed tomography scans in 11.4% of NOM patients at hospital day 6–7 and tended to be associated with high‐grade splenic injuries and continuous symptoms. Repetitive screening of these patients 6–7 days after injury might be warranted because of the potential risk of delayed events.

## Aim

Recently, as the quality of non‐operative management (NOM) has developed, blunt injuries to visceral organs such as spleen, liver, or kidney are tending to be managed non‐operatively with a high rate of success.^1^, ^2^, ^3^ However, NOM of these injuries sometimes leads to critical delayed complications and the possibility of lethal damage for the patients.^4^ Pseudoaneurysm formation and delayed rupture are two of the most critical complications that can occur suddenly and unexpectedly.

Some reports^5^, ^6^ have described the incidence and timing of splenic pseudoaneurysm formation, but there is no global consensus for repetitive imaging with computed tomography (CT), especially for injuries classified as low grade on the Trauma Organ Injury Scale of the American Association for the Surgery of Trauma (AAST), and there are no recommendations for the appropriate duration of hospital admission.^7^, ^8^

The purpose of this study is to examine the incidence and timing of such delayed undesirable events and to establish advisable timing of follow‐up imaging and appropriate duration for observation or disposition of patients with blunt visceral organ injuries.

## Methods

This study was undertaken retrospectively in the Department of Trauma and Critical Care of Osaka City University Hospital (Osaka, Japan). During the period January 2013–January 2017, all patients aged 16 years or older who were admitted to our institution because of blunt liver, kidney, or splenic injuries were included. We reviewed the patients' demographics, injury descriptions, values of laboratory data on admission, volume of blood products used in the 24 h after admission, timing of follow‐up CT scans, management techniques, and outcomes obtained from the patients' medical records.

### Treatment strategies for patients with abdominal visceral injuries

In our institution, the patients who have clinical findings or suspicion of abdominal trauma, if focused abdominal sonography for trauma (FAST) is positive and the patient's unstable hemodynamics makes their transfer to the CT scan room or operating room difficult, we immediately carry out resuscitative surgery in the emergency unit without a pan‐scan CT. If FAST is positive and hemodynamics are stable or controlled, or if FAST is negative, a contrast‐enhanced CT (CECT) scan is carried out to assess the injuries at the time of hospital admission.

If the CECT findings show contrast blush in the arterial phase or pooling in the dynamic phase, we basically plan transcatheter arterial embolization (TAE) to embolize the sites of bleeding, even if the hemodynamic status is stabilized without intervention.

For the patients with an AAST Trauma Organ Injury Scale of grade III–IV or in those treated with TAE, a follow‐up CT scan is undertaken on day 1 or 2 following hospital admission and then approximately 1 week after the injury if clinical symptoms or vital signs remain stable. For the patients with AAST Trauma Organ Injury Scale of grade I–II, the follow‐up CT scan is carried out approximately 1 week after the admission. If the patients have intolerable pain, sudden onset of pain, or positive FAST findings during the observation period, we carry out an extra emergent CT scan.

Including the timing of ambulation, the activity level of the patients after admission is determined essentially on the basis of the clinical symptoms or the results of follow‐up CT scan. The pseudoaneurysm is defined as diameter >7 mm.

### Materials and procedure of TAE in our institution

Transcatheter embolization for trauma patients is always carried out by interventional radiologists. For injuries of visceral organ arteries, we usually use gelatin sponges with concomitant use of pushable coils if needed. The exact procedure depends on the hemodynamic conditions, injured sites, and concomitant injuries of the patients. If the hemodynamic status of the patient is sufficiently stable and there are no concomitant injuries, we often choose distal embolization and selectively embolize the artery as close as possible to the injured sites. If the condition of the patient or the injured sites does not permit a long procedural time or selective embolization, we choose proximal artery embolization for the abbreviated intervention.

### Patient selection

During the study period, 57 patients were diagnosed as having liver, splenic, or kidney injuries. Eighteen patients were excluded as their hemodynamics had not been stabilized and they were treated with immediate laparotomy. We also excluded four patients because of severe brain injury that had not recovered, as indicated by not attaining a Glasgow Coma Scale score >8 during hospital admission, and patients with spinal or pelvic injuries who had needed continuous bedrest. Finally, 35 patients treated with NOM were included in this study. Of the 35 patients, 14 patients underwent TAE; the other 21 patients had no TAE indications and were observed carefully.

### Statistical analysis

All statistical data are presented as median (25–75% interquartile range) or number as the results of statistically non‐normal distribution of the collected data. Non‐parametric numerical data were compared using the Mann–Whitney *U*‐test. A value of *P* < 0.05 was considered statistically significant. Data were analyzed using IBM spss Statistics, version 22 (SPSS, Chicago, IL, USA).

## Results

**Figure 1:**
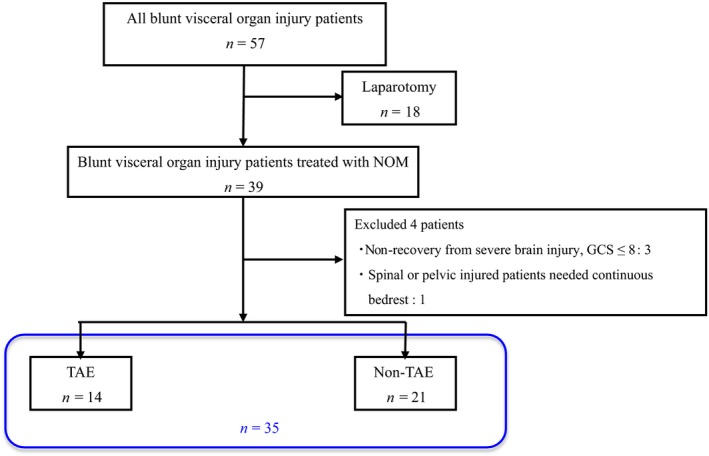
Of 57 patients with blunt visceral organ injury, 18 patients treated with immediate laparotomy were excluded from the study. Four patients with severe brain injury or spinal or pelvis injury who needed continuous bedrest were also excluded. The remaining 35 patients treated with non‐operative management (NOM) were included in this study. GCS, Glasgow Coma Scale; TAE, transcatheter arterial embolization.

During the period January 2013–January 2017, 57 patients with documented blunt splenic (S), liver (L), or kidney (K) injury were admitted in our hospital. Twenty‐two patients were excluded described as above, and 35 patients (L, 10; S, 17; K, 6; L & S, 1; S & K, 1) treated with NOM were included (Fig. 1). Of the 35 patients, 14 patients (L, 4; S, 9; K, 1) were treated with TAE. The patient characteristics, mechanism of injury, and injury descriptions are shown in Table 1.

**Table 1:** Demographic data of patients with blunt visceral organ injury treated with non‐operative management (*n* = 35)

	*n* = 35
Sex, male / female	25/10
Age, years	41.5 (24.0–62.5)
Mechanism of injury	
Motor vehicle accident	22
Fall from height	8
Other unexpected injury	5
Treated with TAE	14
Trauma Organ Injury Scale (I / II / III / IV / V)	
Liver	10 (3/3/4/0/0)
Spleen	17 (2/6/8/1/0)
Kidney	6 (1/3/1/1/0)
Liver & spleen	1 (0/0/1/0/0 & 0/0/1/0/0)
Spleen & kidney	1 (0/0/1/0/0 & 0/0/1/0/0)

TAE, transcatheter arterial embolization.

During the observational period, delayed undesirable events were confirmed in four patients (11.4%). All delayed events were detected in splenic injury, and delayed events occurred in 23.5% of splenic injured patients. As Table 2 shows, splenic pseudoaneurysm was confirmed by CECT on hospital day 6–7 in three patients, and two of them were initially treated with TAE. Delayed splenic rupture was confirmed on hospital day 7 in one patient who had also initially been treated with TAE. The AAST Trauma Organ Injury Scale grades of these patients were III and IV; there was no incidence of delayed undesirable events in the patients with an injury scale grade of I or II during the observation period of this study.

**Table 2:** Patients with blunt visceral organ injury and delayed undesirable events, treated with non‐operative management

Sex	Male	Male	Male	Male
Age, years	36	39	79	48
Mechanism of injury	MVA	MVA	MVA	MVA
Detected delayed event	PA	PA	PA	DR
Trauma Organ Injury Scale	III	III	III	IV
Initial intervention	–	TAE	TAE	TAE
Event detected after admission, days	6	7	6	7

–, not applicable (Only conservation); DR, delayed organ rupture; MVA, motor vehicle accident; PA, pseudoaneurysm; TAE, transcatheter arterial embolization.

As the initial TAE procedure, the lower and middle branches of the splenic artery in two of these four patients were selectively embolized using gelatin sponges and pushable coils. The remaining patients with pseudoaneurysm underwent selective embolization of the lower branch of the splenic artery using gelatin only.

The initial CT scans carried out at the time of admission and the second follow‐up CT scans undertaken 1–2 days after admission did not show any significant findings that could help in predicting the undesirable results of delayed events such as pseudoaneurysm formation or delayed rupture.

The values of laboratory data, lactate, volume of used blood products in the 24 h after admission, and Trauma and Injury Severity Score showed no significant differences between the two groups (Table 3).

**Table 3:** Outcomes of patients with blunt visceral organ injury treated with non‐operative management, grouped according to delayed undesirable events

	Event‐free patients	Delayed‐event patients	*P*‐value
Lactate, mmol/L	2.4 (1.3–3.3)	3.1 (2.4–4.5)	0.18
AST, IU/L	66 (35–206)	117 (41–409)	0.58
ALT, IU/L	40 (16–148)	91.5 (36–117)	0.39
Creatinine, mg/dL	0.73 (0.54–0.91)	0.88 (0.74–1.09)	0.19
Hemoglobin, g/dL	11.7 (9.3–13.1)	13.2 (9.7–16.7)	0.39
PT‐INR	1.09 (1.01–1.15)	1.04 (0.99–1.08)	0.48
Fibrinogen, mg/dL	207(186–291)	228 (116–270)	0.52
FDP, µg/mL	40.5 (16.9–106.1)	22.9 (6.8–98.1)	0.43
Volume of blood products used in 24 h, mL	1040 (0–1840)	1280 (0–3610)	0.26
Time from admission to start of per oral nutrition, days	2.0 (1.0–3.0)	3.0 (1.0–4.0)	0.40
Time from admission to increased activity level, days	2.0 (2.0–3.0)	2.0 (1.0–3.0)	0.54
Duration of continuous symptoms, days	2.0 (1.0–3.0)	6.0 (5.0–7.0)	0.04
TRISS	0.94 (0.912–0.975)	0.91 (0.788–0.970)	0.42

Statistical data are presented as median (25–75% interquartile range) or number.

ALT, alanine aminotransferase; AST, aspartate aminotransferase; FDP, fibrin degradation product; PT‐INR, prothrombin time – international normalized ratio; TRISS, Trauma and Injury Severity Score.

Evaluation of the relationship between patients with continuous intolerable pain using pain control drugs, such as fentanyl, and those who experienced delayed events showed a tendency for longer continuous abdominal pain in those with delayed events than in event‐free patients (*P* = 0.04). Other specific symptoms or complaints were not documented for the patients with delayed events. Vital signs like fever or heart rate did not change to an extent to detect the delayed events for these patients. There were no significant relationships between the starting time of per oral nutrition or early ambulation and the delayed events (Table 3).

All patients with delayed events were treated successfully with TAE and discharged to home with no complications at approximately 2 weeks after the intervention.

## Discussion

The patients treated with NOM for their visceral organ injuries have been increasing, hence the incidence of delayed events should be predicted as much as possible. In this study, delayed events were detected in 11.4% of the patients within 7 days after injury. Previously published papers describing these delayed events are almost all limited to splenic pseudoaneurysm. Davis *et al*.^5^ and Weinberg *et al*.^6^ reported the incidence of splenic delayed events of 7.7% and 7.1%, respectively, in patients with blunt splenic injury treated with NOM. Compared to these reports, the incidence rate of splenic delayed events in this study tended to be high (21.1%). We consider that this is resulted from differences in the severity of injury. As Table 1 shows, 57.9% of the patients with splenic injury had AIS grade III injury, and thus the incidence rate of delayed undesirable events turned out to be high percentage of total splenic injury. The other possibilities that should be considered were technical issues with transcatheter embolization. We always have to be careful about preserving organs, especially in young patients, but selective embolization under the status of relatively low blood pressure carries high risks of incomplete embolization because of arterial spasm or unidentified injured arteries, and following delayed events like pseudoaneurysm formation. Describing the timing of delayed splenic events, Leeper *et al*.^9^ recently reported their experience of 12 years with the management of hemodynamically stable blunt splenic injuries. Delayed development of pseudoaneurysm or arterial extravasation occurred in only 6% of the patients on follow‐up CT scan 48 h after injury. The delayed events occurring in our four patients were detected approximately 7 days after injury. Muroya *et al*.^10^ also reported that the detection of delayed events by follow‐up CT scan were mostly at an interval of 1–8 hospital days after injury.

Francisco *et al*.^11^ reported that post‐traumatic hepatic artery pseudoaneurysm or delayed rupture was uncommon and occurred in only approximately 1% of hepatic trauma cases. As treatment of liver injury with NOM has increased, major complications such as delayed bleeding, bile leakage, hepatic necrosis, gallbladder necrosis, abscesses, or vessel fistulae are also being recognized increasingly, and these events were reported to occur in approximately 12–24% of NOM patients.^12^, ^13^, ^14^ Fortunately, these complications can often be recognized from the patient's vital signs, physical examination, and laboratory studies.

Although renal artery pseudoaneurysm is also rare, Yamaçake *et al*.^15^ reported in a case report series that it was possible for a pseudoaneurysm to develop from blunt renal trauma even years after the initial injury. We found no hepatic or renal delayed complications during this study period, but we should continue to carefully follow up these patients based on the findings of these previous reports.

The relationships between symptoms or the timing of starting per oral nutrition and delayed events remain unclear. Bukur *et al*.^16^ reported that in patients with blunt renal injury and an AAST Trauma Organ Injury Scale ≤grade III who developed delayed complications, all were symptomatic, and they suggested that selective follow‐up imaging should be based on laboratory and clinical signs. In addition, there is no evidence that per oral nutrition should be withheld in patients with visceral organ injuries; we also found no relationship between the starting day of nutrition intake and delayed events.

Although activity level is one of the most important problems for NOM patients, there are few data on the activity level permitted following injury. London *et al*.^17^ retrospectively reviewed time to mobility in patients with various solid organ injuries and reported that early mobilization did not correlate with a higher rate of NOM failure. We also found no significant relationship between early ambulation of the patient and delayed events. However, Peitzman *et al*.^18^ recommended a period of 4 days of careful observation under monitoring as most NOM failures occur within the first 4 days.

Although routine CT follow‐up has recently tended not to be recommended, especially in patients with a low‐grade organ injury score, the findings from this study and previous reports suggest that monitoring and the timing of follow‐up CT scan 6–7 days after injury could be a reasonable period to detect delayed undesirable events stemming from blunt visceral organ injuries.

## Limitations

The present single‐center study is a small preliminary report, and we will definitely have to plan further multi‐institutional, prospective, randomized trials on the basis of this study to assess the appropriate timing of follow‐up CT scans.

## Conclusions

Delayed undesirable events were recognized to occur during hospital days 6–7 by follow‐up CT in 11.4% of patients treated with NOM. These events tended to be associated with high‐grade splenic injuries and continuous symptoms. We conclude that repeated screening of these patients approximately 6–7 days after injury may be warranted because of the potential risk of delayed events occurring within this time.

## Disclosure

Conflict of interest: None declared.
